# Outcome of patients with HIV-related germ cell tumours: a case–control study

**DOI:** 10.1038/sj.bjc.6601762

**Published:** 2004-03-30

**Authors:** T Powles, M Bower, J Shamash, J Stebbing, J Ong, G Daugaard, A De Ruiter, M Johnson, M Fisher, J Anderson, M Nelson, B Gazzard, T Oliver

**Affiliations:** 1 Department of Oncology, Chelsea and Westminster Hospital, Fulham Road, London SW10 9NH, UK; 2 Department of HIV Medicine, Chelsea and Westminster Hospital, London, UK; 3 St Bartholomew's & Royal London Hospital, London, UK; 4 Rigshospitalet, Copenhagen, Denmark; 5 Guys and St Thomas Hospital, London, UK; 6 Royal Free Hospital, London, UK; 7 Royal Sussex County Hospital, Brighton, UK

**Keywords:** HIV, testicular cancer, germ cell tumour, HAART, survival

## Abstract

Testicular germ cell tumour (GCT) is not an AIDS-defining illness despite an increased incidence in men with HIV infection. We performed a matched case-control study comparing outcomes in HIV-positive men and the general population with GCT, using three age and stage matched controls for each case. There was no difference in the 5-year GCT-free survival between cases and controls. However, overall survival was significantly decreased in the cases (log rank *P*=0.03). HIV was responsible for 70% of this mortality. The relapse-free survival for stage I patients treated with orchidectomy and surveillance was not affected by HIV status (log rank *P*=0.68). There was no difference in disease free survival in patients with metastatic disease (log rank *P*=0.78). The overall survival has not improved since the introduction of highly active antiretroviral therapy (log rank *P*=0.4). Thus, HIV-related GCT is not more aggressive than GCT in the general population.

Although non-Hodgkin's lymphoma (NHL) and Kaposi's sarcoma account for the vast majority of HIV-1-associated malignancies, other tumours, such as germ cell tumours (GCT), also occur more frequently in HIV-1 infection (Logothetis *et al*, 1985; Gabutti *et al*, 1995; Goedert *et al*, 1998; Lyter *et al*, 1995; Dal Maso *et al*, 2001; Frisch *et al*, 2001). These non-AIDS-defining tumours are relatively rare, and published series have been small. Therefore, accurately assessing the outcome of these patients and the influence of HIV on the course of GCT have been difficult (Damstrup *et al*, 1989; Moyle *et al*, 1991; Bernardi *et al*, 1995; Timmerman *et al*, 1995; Hentrich *et al*, 1996; Fizazi *et al*, 2001).

Our group recently reported a large series of HIV-1-related GCT (Powles *et al*, 2003a). It showed that these tumours occur more frequently in HIV-1, and that highly active antiretroviral therapy (HAART) has not resulted in a major change in the incidence of the disease. Moreover, patients had a good outcome with respect to the GCT, and the majority of the mortality in this cohort related to HIV-1 infection.

Unfortunately, like other previously published series, our study did not directly compare HIV-positive and -negative individuals, and therefore uncertainty surrounding the relative outcome of these patients remains. This is especially true in three subgroups of patients with HIV-related GCT.

The first group of patients are those with stage I HIV-related GCT who are treated with orchidectomy and surveillance alone. It has been found that tumour infiltrating lymphocytes may improve the prognosis in stage I GCT managed by surveillance (Nakanoma *et al*, 1992; Parker *et al*, 2002). Hence, it is postulated that HIV patients may be at increased risk of relapse of disease due to lack of immune surveillance, and it is currently not known if their outcome is the same as HIV-negative men.

The second area of uncertainty relates to HIV-positive patients with metastatic GCT. There is some speculation that patients with HIV-1 have increased intrinsic resistance to chemotherapy, despite HAART and similar treatment regimens. These data come from patients with HIV-related NHL, and is thought to involve high levels of expression of the multidrug-resistant gene (MDR-1) that encodes the p-glycoprotein efflux pump (Little *et al*, 2000; Tulpule *et al*, 2002). Currently, there is no data comparing the outcome of HIV-positive and -negative patients with metastatic GCT.

The final area of ambiguity relates to the impact of HAART on the survival of patients with GCT. It appears that HAART has a beneficial effect on the outcome of HIV patients with other malignancies (Bower *et al*, 1999; Ribera *et al*, 2002; Tam *et al*, 2002; Gerard *et al*, 2003; Powles *et al*, 2003b); however, this has not been shown in patients with GCT.

Therefore, in this study, we compared the treatment outcomes for HIV-positive and -negative men with GCT, with particular emphasis on patients with stage I disease treated with orchidectomy and surveillance, as well as the survival of patients with metastatic disease. We also examined the effects of HAART on the response to chemotherapy and overall survival.

## PATIENTS AND METHODS

The clinical details of patients with HIV-related GCT, diagnosed since the beginning of the HIV epidemic in 1983, were retrieved from prospective data bases from six HIV centres Chelsea and Westminster Hospital (London, UK), Rigshospitalet Hospital (Copenhagen, Denmark), St Bartholomew's and Royal London Hospital (London), Royal Free Hospital (London), Royal Sussex County Hospital (Brighton) and Guys and St Thomas' Hospital (London). The HIV-negative control group was derived from a large prospective cohort from one treatment centre (St Bartholomew's and Royal London Hospital), and only men diagnosed after 1983 were selected.

For each HIV-positive case patient, three HIV-negative controls were identified who were matched for stage, histology, age (within 5 years of the case patient) and date of diagnosis (within 5 years of the case patient). Patients diagnosed prior to 1 January 1996 were defined as being in the pre-HAART era, while those diagnosed after this date were ascribed to the post-HAART era.

All patients had histologically confirmed GCT. Staging was classified using the American Joint Committee on Cancer staging system (AJCC, 1997). Patients with metastatic disease were also classified using the International Germ Cell Cancer Collaboration Group (IGCCCG) prognostic scoring scheme, which divides metastatic GCT into good, intermediate and poor prognosis groups (IGCCCG, 1997). Response to treatment and toxicity was evaluated using the RECIST guidelines and the National Cancer Institute common toxicity criteria (CTC) respectively (Therasse *et al*, 2000). Disease-free survival and overall survival were plotted according to the Kaplan–Meier method (Kaplan and Meier, 1958).

## RESULTS

In all, 35 were with HIV-related GCT were identified and matched with 105 HIV-negative controls. The patient characteristics are described in Table 1

**Table 1:** Characteristics and outcome of patients with GCT

	**HIV +ve**	**HIV −ve**
Number	35	105
Median age (range)	34 years (27–64)	33 years (25–64 years)
Number with seminoma	26 (74%)	78 (74%)
Number with metastatic disease	14 (40%)	42 (40%)
Median follow-up (range)	4.6 years (0.3–14)	4.3 years (0.1–17.2)
GCT-related deaths	3 (9%)	11(10%)
Other deaths (including HIV)*	7 (20%)	0

* *P*<0.01. GCT=germ cell tumour.

**Figure 1:**
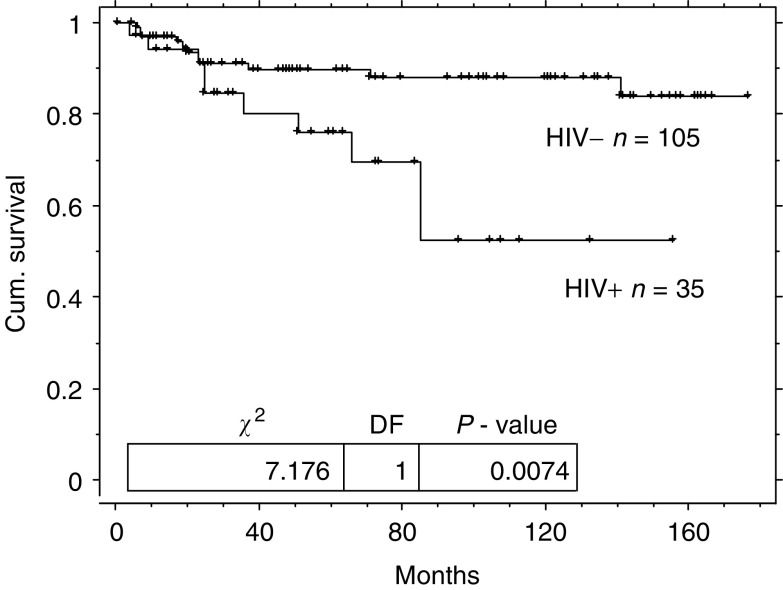
Kaplan–Meier overall survival duration curve from diagnosis of GCT for patients with HIV infection and matched controls.

. The overall survival of the HIV-positive cohort was significantly decreased when compared to the HIV-negative group (log rank *P*=0.0077). The 2- and 5-year actuarial survivals are 94% (95% CI: 86–100) and 76% (95% CI: 60–2) for the HIV-positive patients compared to 94% (95% CI: 90–98) and 90% (95% CI: 84–96) for the HIV-negative group. However, there is no difference in the disease-free survival between the two groups (log rank *P*=0.8). The 2- and 5-year actuarial disease-free survivals are 82% (95% CI: 69–95) and 82% (95% CI: 69–95) for the HIV-positive patients compared to 82% (95% CI: 74–90) and 80% (95% CI: 72–88) for the HIV-negative group, see Table 1, Figures 1

and 2

**Figure 2:**
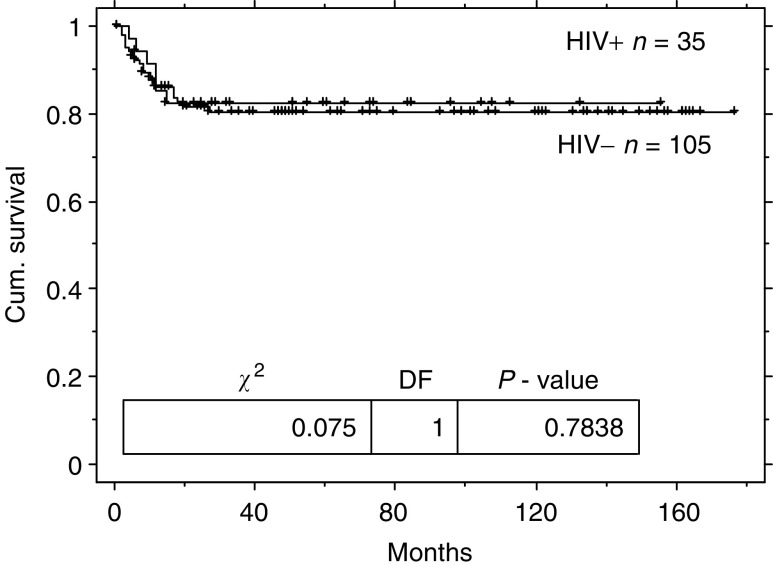
Kaplan–Meier germ cell cancer-free survival duration curve from diagnosis of GCT for patients with HIV infection and matched controls.

.

### Effect of HAART on survival

**Figure 3:**
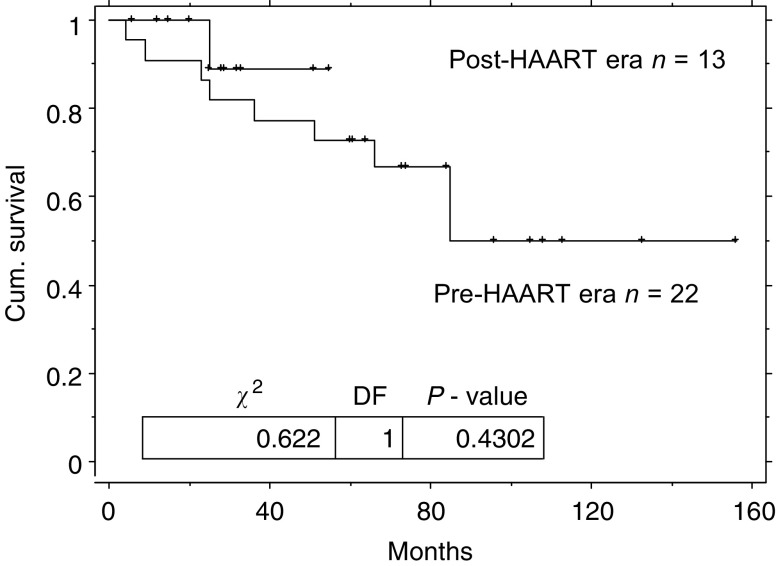
Kaplan–Meier overall survival duration curve from diagnosis of GCT for patients with HIV infection comparing patients who presented in the pre- and the post-HAART eras.

In total, 21 patients were diagnosed with GCT before the introduction of HAART in 1996 and 14 were diagnosed after this date. Patients in the pre-HAART era had a worse overall survival than the control population (log rank *P*=0.03, see Figure 3

). The 2- and 5-year actuarial survivals are 91% (95% CI: 79–100) and 73% (95% CI: 54–92) for the HIV-positive patients in the pre-HAART era. However, since the introduction of HAART, the outcome has improved due to a decrease in HIV-related deaths (seven in the pre-HAART era and none in the post-HAART era, *P*=0.03), there were a similar number of tumour-related deaths in the pre- and post-HAART eras (2 and 1, respectively, *P*=0.7). The 2- and 5-year actuarial survivals are 100 and 88% (95% CI: 66–100) for the HIV-positive patients in the post-HAART era.

### Patients with metastatic disease

In all, 14 patients presented with metastatic disease and their characteristics are described in Table 2

**Table 2:** Characteristics and outcome of patients with metastatic GCT

	**HIV +ve**	**HIV –ve**
Number	14	42
Median age (range)	36 years (27–44 years)	31 years (21–53 years)
Median follow-up (range)	4.6 years (0.3–9 years)	4.3 years (0.1–13.2 years)
		
*IGCCCG prognostic classification*		
Good	8 (57%)	27 (57%)
Medium	5 (36%)	15 (36%)
Poor	1 (7%)	5 (7%)
		
GCT-related deaths	2 (14%)	9 (21%)
HIV-related deaths	2 (14%)	0

GCT=germ cell tumour; IGCCCG-International Germ Cell Cancer Collaboration Group.

. All but one were treated with curative intent, using standard platinum-based combination chemotherapy, see Table 3

**Table 3:** Treatment details for patients with metastatic HIV-related GCT

**Regimen**	**Number of patients**
BEP X3	5
BEP X4	3
BEP X5	1
EP X4	3
Radiotherapy (40 Gy in 20#)	1

BEP=bleomycin, etoposide and cisplatin in 3 weekly cycles; EP=Etoposide and cisplatin in 3 weekly cycles; GCT=germ cell tumours.

**Figure 4:**
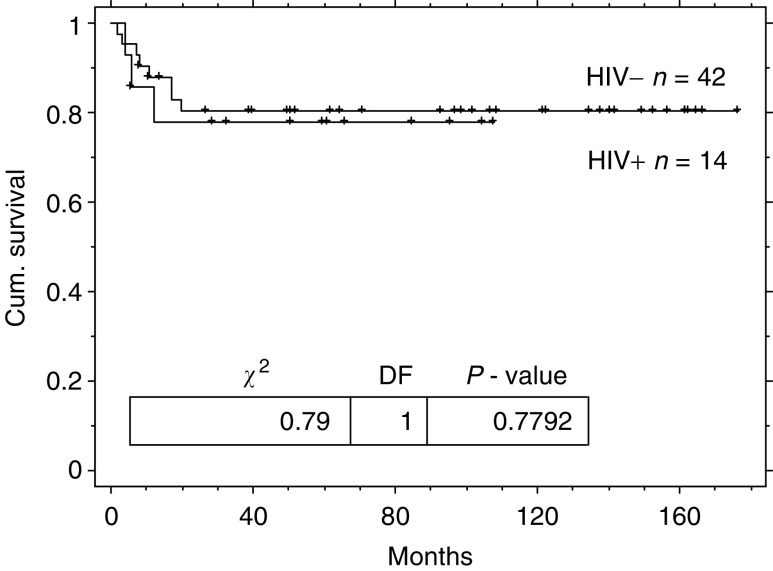
Kaplan–Meier overall relapse-free survival duration curve from diagnosis of metastatic GCT for patients with HIV infection and matched controls.

. The remaining patient had advanced HIV disease in the pre-HAART era and declined treatment. Two (15%) patients required a dose reduction (one due to myelosuppression and one because of nephrotoxicity). Two (15%) other patients suffered grade 3–4 myelosuppression and two (15%) developed grade 3–4 neurotoxicity. The toxicity details are not available for the HIV-negative cohort and therefore cannot be compared directly. The GCT-free survival of the HIV-positive and -negative groups was not significantly different (log rank *P*=0.78) see Table 2 and Figure 4

. The 2- year disease-free survivals for patients with metastatic GCT are 77% (95% CI: 55–99) for those who are HIV seropositive and 80% (95% CI: 68–92) for those who are HIV seronegative.

### Stage I patients managed with orchidectomy and surveillance

Of the 21 (81%) HIV-positive patients with stage I GCT, 17 were treated with orchidectomy and surveillance alone. The details of these patients are shown in Table 4

**Table 4:** Characteristics and outcome of patients with stage I GCT treated with orchidectomy surveillance

	**HIV +ve**	**HIV –ve**
Number	16	48
Median age (range)	33.5 years (27–64 years)	33.5 years (25–64 years)
Median follow-up (range)	3.0 years (1–13 years)	3.8 years (0.1–14.6 years)
Relapses of GCT	4 (25%)	12 (25%)
GCT-related deaths	1 (6%)	1 (1%)
Other deaths (including HIV)*	3 (20%)	0

This table does not include the five HIV-positive and 15 HIV-negative patients with stage I disease that received adjuvant treatment. (GCT=germ cell tumour).

* *P*>0.05.

**Figure 5:**
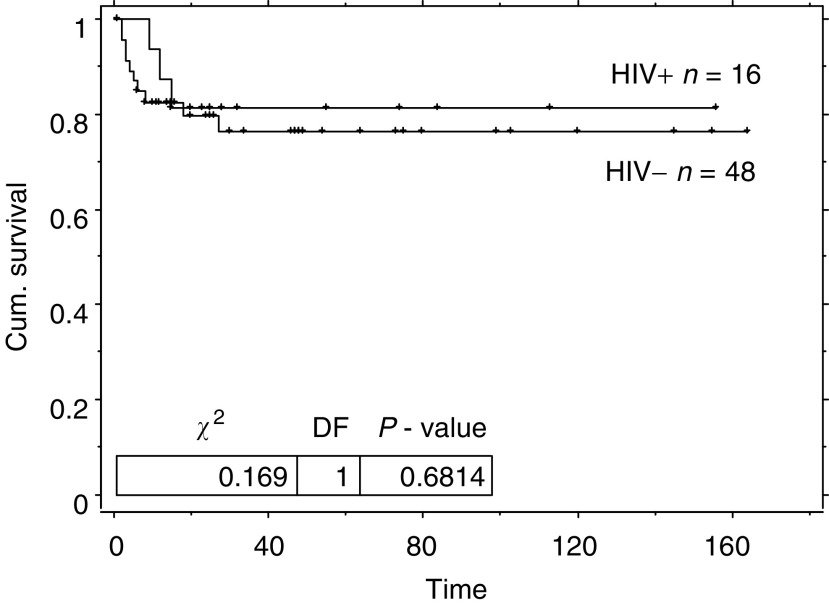
Kaplan–Meier relapse-free survival duration curve from diagnosis of stage I GCT for patients with HIV infection and matched controls managed by orchidectomy and surveillance.

. The GCT relapse-free survival of this group was not different to that of the matched HIV-negative population (log rank *P*=0.91) see Table 4 and Figure 5

. The 2-year disease-free survivals for patients with stage I disease managed by orchidectomy and surveillance are 81% (95% CI: 63–100) for HIV-positive men and 79% (95% CI: 67–91) for HIV-negative men.

### Other patients with stage I HIV-related GCT

The remaining five patients with stage I disease were treated with orchidectomy and adjuvant radiotherapy (4) or chemotherapy (1). Once again, standard regimens designed for HIV negatives were used. None of these patients have relapsed or died from GCT-related disease.

## CONCLUSIONS

Initial studies suggested that patients with HIV-related cancers (Kaposi's sarcoma, NHL and cervical cancer) had a worse outcome compared to HIV-negative controls for three reasons. Firstly, the HIV itself caused much of the mortality often due opportunistic infections such as *Pneumocystis carinii* pneumonia (Bernardi *et al*, 1995). Secondly, it was speculated that patients with HIV infection tolerated chemotherapy less well resulting in dose reductions that entailed lower response rates (Wilson *et al*, 1992; Hentrich *et al*, 1996). The third possible reason for the decreased survival was due to more aggressive tumours in this group or intrinsic chemotherapy resistance in patients with HIV (Tessler and Catanese, 1987; Sridhar *et al*, 1992; Vyzula and Remick, 1996).

Since the introduction of HAART, HIV-related mortality has decreases and the outcome of patients with Kaposi's sarcoma and lymphomas have improved (Tam *et al*, 2002). We have also shown that the survival of HIV-positive patients with lung cancer is not significantly different to controls (Powles *et al*, 2003b). This improved survival is thought to be due to not only decreased HIV related mortality but also better tolerance of chemotherapy (Sparano *et al*, 1998; Esdaile *et al*, 2002). However, for patients with AIDS-related lymphoma, the problem of intrinsic chemotherapy resistance persists (Little *et al*, 2000). One of the mechanisms of increased intrinsic chemoresistance relates to over activity of the p-glycoprotein efflux pump, which is associated with MDR-1 over expression, and occurs three times more frequently in AIDS-related lymphoma than in lymphomas in the immunocompetent population (Tulpule *et al*, 2002).

We demonstrate here for the first time that HIV-positive patients with metastatic GCT have a similar disease-free survival compared with HIV-negative controls. Our patients, with the exception of one man who declined chemotherapy, were treated with curative intent. These findings imply that unlike AIDS-related lymphomas, HIV-related GCT is not an intrinsically chemoresistant tumour.

Treatment toxicity in the HIV-positive and -negative cohorts was not directly compared; however, there were no treatment-related deaths in either group. This and other studies have suggested that the HIV-positive group do not have excess toxicity compared to historical HIV-negative controls (Williams *et al*, 1987; Timmerman *et al*, 1995; Hentrich *et al*, 1996). The detrimental effect of chemotherapy on immune parameters has been described previously in this group, overall it is associated with a 30% fall in the CD4 cell count (Powles *et al*, 2003a)

HIV-negative patients with GCT localised to the testis are initially treated with orchidectomy. Further management of these patients is controversial. Retroperitoneal lymph node dissection, adjuvant chemotherapy and adjuvant radiotherapy are all established treatment options. However, these patients may be managed with surveillance alone, and despite a relapse rate of approximately 30%, their overall survival is the same as patients who received adjuvant treatment (Francis *et al*, 2000). To date, the recommended treatment for stage I HIV-related GCT has been orchidectomy and radiotherapy or retroperitoneal lymph node dissection (Hentrich *et al*, 1996). This is in part due to a fear that these patients may be at a higher risk of relapse and that metastatic disease may be associated with a worse outcome compared to HIV negative controls (Tessler and Catanese, 1987; Wilson *et al*, 1992). The data presented here show that orchidectomy and surveillance and Catanese, do not result in an increased relapse rate in these patients compared to the HIV-negative population, and that this is a safe management policy without the potentially immunosuppressive adjuvant therapy. This is of particular importance in the HIV population as they are more likely to succumb to HIV infection than recurrent GCT. Patients on a surveillance programme must be competent and fully compliant with the protocol to ensure the safety of this regimen. Reports suggest intravenous drug abusers comply poorly with treatment and are often lost to follow-up (Bernardi *et al*, 1995). It is therefore reasonable to encourage these patients to have some form of adjuvant treatment in order to reduce the risk of relapse.

The overall survival of patients with HIV-related GCT was worse than HIV-negative controls in the pre-HAART era. The poor outcome in this cohort was due to HIV-related disease, which was responsible for seven of the nine deaths during this period. Since the introduction of HAART fewer of these patients have died. This is due to a decrease in HIV-associated complication rather than improved GCT survival, with no HIV-related deaths in the post-HAART era.

Previous studies have suggested ‘tailor made’ treatment for patients with HIV related GCT in order to avoid potential interactions and side effects associated with HIV and its treatment (Wilson *et al*, 1992; Hentrich *et al*, 1996) However, this and other studies suggest that patients tolerate standard treatment remarkable well, and should be treated in an similar manner to HIV negatives (Bernardi *et al*, 1995; Timmerman *et al*, 1995). To date, no treatment-related deaths have been reported for an HIV-related GCT patient. Cytotoxicity chemotherapy and radiotherapy have a suppressive effect on immune parameters. Despite this, none of the patients developed opportunistic infections in this or the other large published series (Bernardi *et al*, 1995). This emphasises the need for oncologists to work in conjunction with HIV physicians with regard to commencing HAART and prophylaxis against opportunistic infections.

In conclusion, it appears that patients with HIV-related GCT have identical response rates and tumour-free survival compared to HIV-negative controls. However, they have a decreased overall survival emphasising the need for outcome studies such as this to acquire information with regard to this end point.

Before the introduction of HAART, HIV was responsible for much of the mortality of this group that has now reduced. Orchidectomy and surveillance is a safe alternative to other forms of adjuvant treatment and is not associated with an increased risk of relapse. Unlike other HIV-related malignancies, GCT are not intrinsically chemoresistant and have a similar response rate and disease-free survival compared to HIV-negative controls.
